# The Intraoperative Role of Artificial Intelligence Within General Surgery: A Systematic Review

**DOI:** 10.7759/cureus.73006

**Published:** 2024-11-04

**Authors:** Deema Othman, Ahmad Kaleem

**Affiliations:** 1 College of Medicine, Mohammed Bin Rashid University of Medicine and Health Sciences, Dubai, ARE; 2 General Surgery, Mediclinic Parkview Hospital, Dubai, ARE

**Keywords:** artificial intelligence, deep learning, general surgery, intraoperative, medical education

## Abstract

The role of artificial intelligence has been explored in many industries across the world. The medical field is no exception with studies regarding its use for development of algorithms in cancer screening and its diagnostic utility in clinical radiology. This study aims to review current literature on intraoperative use of artificial intelligence within general surgery to identify the latest developments, the major challenges and the trajectory of this field. A literature search was done on PubMed on May 28, 2024, using the terms: ((artificial intelligence) AND (general surgery)). Only publications in English and studies involving human subjects were considered. Exclusion criteria included duplicate papers, irrelevant titles, abstracts, themes, and non-English papers. A literature search on PubMed yielded 13 relevant articles. Among these, five articles focused on intraoperative guidance, four addressed surgical education and training, and four were survey-based exploring perceptions regarding artificial intelligence. Key themes included the development of artificial intelligence-based autonomous actions during surgery and its role in enhancing surgical training. Limitations identified included restricted data availability, ethical concerns, and a lack of validation tools, which pose significant obstacles to progress in this area. Despite existing limitations, the potential for integrating artificial intelligence into general surgery is promising. Careful attention is needed to overcome challenges and maximize its benefits.

## Introduction and background

Artificial intelligence (AI) is a rapidly expanding domain across all fields, raising numerous questions about its capabilities and limitations. These questions are particularly prominent in the field of medicine where AI has been incorprorated in multiple aspects including cancer screening, clinical radiology and to develop algorithms to guide patient management within emergency medicine [1-5]. Studies are rapidly emerging including systematic reviews such as the RAISE study (Radiology Artificial Intelligence: A Systematic Review and Evaluation of Methods) evaluating current literature on AI in radiology [6]. Although AI has been introduced to general surgery, it generally lags behind other fields in terms of both literature and clinical application. A similar delay was also noted with the development of robotic surgery where it was initially hypothesized in the 1960s and actual use began in the late 1980s, eventually becoming accepted as a standard of care [7]. Most developments regarding the use of AI in general surgery have focused on the preoperative and postoperative phases, with algorithms designed to determine the appropriate course of action. However, there is limited knowledge about the application of AI in the intraoperative setting. This study aims to review the current literature on this topic.

## Review

Methods

This article is designed according to the Preferred Reporting Items for Systematic Reviews and Meta-Analyses (PRISMA) guidelines to analyze the available evidence for the intraoperative use of AI in general surgery. A review of full-text articles published in the last five years was conducted, retrieving data from PubMed. The database was searched on May 28, 2024, using the terms: ((artificial intelligence) AND (general surgery)). A total of 1233 records were identified. Only publications in English and studies involving human subjects were considered. Exclusion criteria included duplicate papers, irrelevant titles, abstracts, themes, and non-English papers. Irrelevant titles, abstracts and themes included articles that were related to subsurgical specialties or obstetrics and gynecology rather than general surgery. Furthermore, we excluded articles that only included AI in endoscopy rather than surgery. 

Results

**Figure 1:**
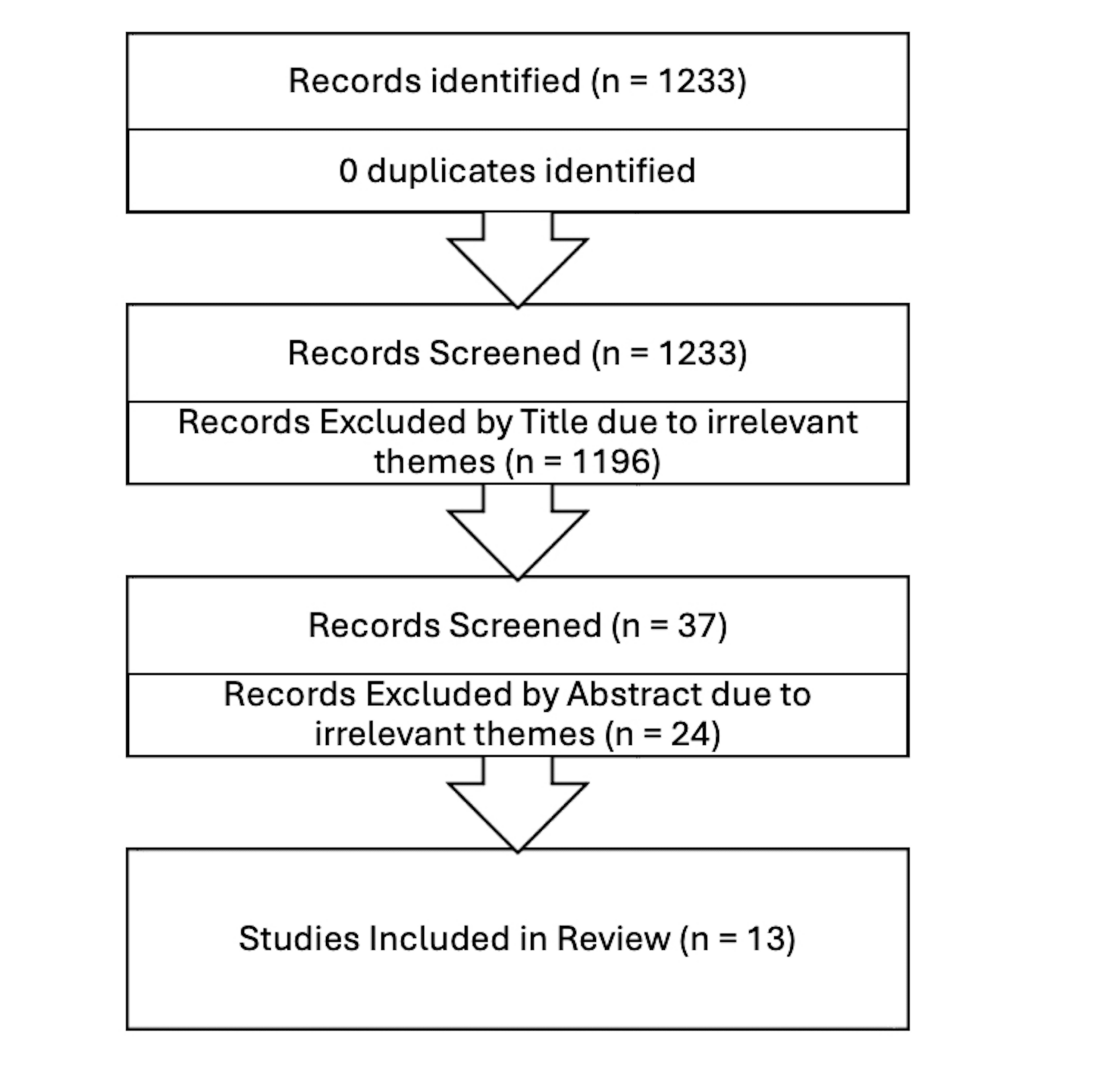
Flow chart of the study selection process according to the Preferred Reporting Items for Systematic Reviews and Meta-analyses (PRISMA) 2020 statement.

Two reviewers screened the 1233 records, ultimately identifying 13 articles for review, as depicted in Figure 1. The results can be appreciated in Table 1.

**Table 1:** Literature Review on Artificial Intelligence in General Surgery Key themes identified included the development of artificial intelligence-based autonomous actions during surgery and its role in enhancing surgical training while limitations included restricted data availability, ethical concerns, and a lack of validation tools. ML = machine learning, DL = deep learning, CV = computer vision, NLP = natural language processing

Year	Article	Author	Research Method	Themes Identified
2021	Artificial Intelligence Surgery: How Do We Get to Autonomous Actions in Surgery? [8]	Gumbs et al.	Review article evaluating challenges in establishing autonomous actions in surgery	Use of AI depends on a complex interplay of ML, DL, CV and NLP: requires compiling large volumes of fully annotated videos capturing each surgical procedure. Surgeons must gain an understanding of the basics of AI to allow for better incorporation into surgical practice in the future.
2022	Current Applications of Artificial Intelligence in Bariatric Surgery [9]	Bellini et al.	Literature review including 36 articles exploring use of machine learning algorithms in bariatric surgery.	Only 3 articles about the intraoperative phase: AI was able to superior to a compartmental model in describing high-resolution pharmacokinetic data of propfolol AI was able to estimate remaining surgery duration using only visual information from laparoscopic videos of cholecystectomies and gastric bypass: significantly outperformed a traditional method of estimating RSD AI was able to automatically identify operative steps in laparoscopic sleeve gastrectomy with a high degree of accuracy
2022	Artificial Intelligence for Intraoperative Guidance: Using Semantic Segmentation to Identify Surgical Anatomy During Laparoscopic Cholecystectomy [10]	Madani et al.	Comparison of AI predictions of safe and dangerous zones during laparoscopic cholectstectomy with expert surgeons	DL can be used to identify safe and dangerous zones of dissection and other anatomical structures in the surgical field during laparoscopic cholecystectomy with a high degree of performance. Safety and use in real-time still needs to be investigated.
2022	Artificial intelligence in surgery: the emergency surgeon’s perspective (the ARIES project) [11]	Simone et al.	Survey assessing trauma surgeons’ knowledge and perspective on AI in surgery.	The application of AI in emergency and trauma surgery is still underway but requires education, accessibility and research funds. Emergency surgeons are willing to contribute to aid in the development of those algorithms.
2021	A systematic review on artificial intelligence in robot-assisted surgery [12]	Moglia et al.	Literature review including 35 articles in multiple specialties	1 study on intraoperative use: AI achieved 75.7% accuracy on recognition of the next surgical task during robot-assisted partial nephrectomy. There is no proof that currently AI can identify the critical tasks of robot-assisted surgery operations.
2023	Executive summary of the artificial intelligence in surgery series [13]	Loftus et al.	Systemic review: 4 studies about the intraoperative use of AI	Use of ML to predict surgeon experience Use of CV algorithms to identify suturing gestures and provide objective feedback ML using video data from multiple operating rooms sources to generate skill assessments in virtual reality Using CV to accurately identify instruments and operative steps using video data
2021	Supporting laparoscopic general surgery training with digital technology: The United Kingdom and Ireland paradigm [14]	Humm et al.	Review aiming to critically evaluate key issues in laparoscopic general surgical training and the role of digital technology.	AI networks trained using expert annotation have been demonstrated to predict the intraoperative phase in laparoscopic cholecystectomy video with an accuracy of 88.9% ± 7.5%, sleeve gastrectomy; 82% ± 4% videos and laparoscopic left sided colorectal resections; 81%. AI was able to recognize and track laparoscopic instruments. The analysis of instrument kinematics can enable interpretation of surgical skill and may be used to develop softwares assessing surgical skills and to identify surgical errors.
2023	Artificial Intelligence Methods and Artificial Intelligence-Enabled Metrics for Surgical Education: A Multidisciplinary Consensus [15]	Vedula et al.	Survey with 40 participants, 17 were surgeons, 11 were engineers or individuals with technical expertise in machine learning/AI	They found that: 2 year time frame: AI should be able to recognize anatomy in the surgical videos and provide immediate performance feedback 5 year time frame: identify parts of the operation where the surgeon needs feedback, overlay images to display surrounding anatomy, guide surgeons on exposure of the surgical field, guide surgenons on optimal use of instruments/devices. 10 year time frame: Enable intraoperative navigability using video, kinematics, and other imaging data for multiple procedures, detect intraoperative error, provide guidance on the next best step to address an intraoperative error or complication, grade difficulty of surgical procedure.
2020	The Virtual Operative Assistant: An explainable artificial intelligence tool for simulation-based training in surgery and medicine [16]	Mirchi et al	50 participants: 28 skilled and 22 novice performed a virtual reality subpial brain tumor resection task.	AI successfully classified skilled and novice participants using 4 metrics with an accuracy, specificity and sensitivity of 92, 82 and 100%, respectively. 4 novices were mistaken for skilled but not the other way around.
2021	Digital surgery for gastroenterological diseases [17]	Hardy et al.	Editorial piece	Subspecialties of surgery have embraced AI for preplanning and intraoperative navigation aid, it is still in its infancy when it comes to general surgery: lack of fixed, easily identifiable landmarks and the tendency for the operator to completely shift the anatomical field intraoperatively. Machine learning requires millions of data points which is found in pre-operative phase with imaging but not as many intraoperative videos exist.
2023	Artificial intelligence in surgical education and training: opportunities, challenges, and ethical considerations – correspondence [18]	Satapathy et al.	Correspondance	Opportunities: personalized learning, simulaton based training, predictive modeling, augmented reality, remote training Challenges: data privacy and security, bias and discrimination, lack of regulatory framework, overreliance on technology, cost Ethical considerations: informed consent, privacy and confidentiality, bias, standardization, responsibility
2022	Robotic Surgery in Rectal Cancer: Potential, Challenges, and Opportunities [19]	Liu et al.	Review article	This is the new era of robot-guided rather than robot-assisted surgery Through reinforcement learning, AI can provide high-quality support in the decision-making process of the surgeon. The most pressing problem in the future of AI is ensuring data privacy and confidentiality.
2022	Robust hand tracking for surgical telestration [20]	Muller et al.	Real-time hand tracking pipeline specifically designed for the application of surgical telestration	Successful localization rate of 98%, averaged over all samples. Compared to the baseline method, this corresponds to a relative improvement of 63% [detection] and 39% [regression]. Furthermore, it performed better in the presence of colored gloves than the baseline.

Within our 13 articles, three were experimental studies [10,16,20], six were review articles [8,9,12-14,19], two were original articles based on surveys [11,15] and two were opinion pieces [17,18]. This is expected due to the novelty of the field. Despite the search including records from the past five years, most articles identified were from the last three years, with only one from 2020 [16].

There were multiple themes identified within the articles regarding the use of AI intraoperatively: autonomus action of AI, surgical education using AI, opinions on the role of AI intraoperatively as well as the ethical considerations in the development of AI.

Autonomus Action of AI

Few studies explored the possibility of developing autonomous action, while most focused on real-time guidance [8-10,12,14,15,19,20]. The study by Bellini et al. included 36 articles, among which one by Hashimoto showed that AI could automatically identify operative steps in laparoscopic sleeve gastrectomy, achieving a mean classification accuracy of 82% ± 4% [21]. In the same review, Twinanda et al. demonstrated that deep learning, using only visual information from laparoscopic videos of 120 cholecystectomies and 170 gastric bypasses, enabled AI to estimate remaining surgery duration with an accuracy outperforming a traditional method of estimating remaining surgery time [22].

Madani et al. utilized deep learning models to identify safe and dangerous dissection zones-specifically, the liver, gallbladder, and hepatocystic triangle-during laparoscopic cholecystectomy. AI predictions were evaluated against annotations by expert surgeons, with intersection-over-union and F1 scores (validated spatial correlation indices) calculated, suggesting that AI could augment surgical performance [10].

Humm et al. demonstrated that the recognition and tracking of laparosocpic instruments has been achieved [14,23]. Furthermore, in the experimental study by Muller et al., AI was able to track hand movements with a successful localization rate of 98%. Minor adjustments, such as colored gloves improved the performance of the AI in tracking hand movements [20].

The study by Moglia et al. reported an accuracy of 75.7% in recognizing the next surgical task during robot-assisted partial nephrectomy [12].

Surgical Education Using AI

Another common theme was the use of AI in surgical education in multiple ways.

In the study by Mirchi et al., 50 participants from a neurosurgery program performed a virtual reality subpial brain tumor resection task. The AI was tasked with classifying the participants into a skilled group and a novice group. It was able to successfully classify skilled and novice participants with an accuracy, specificity and sensitivity of 92%, 82% and 100%, respectively [16].

Moglia et al. demonstrated that AI can recognize the next surgical task by analysing instrument kinematics, suggesting future applications for interpreting surgical skill and identifying errors. This could lead to software development to assess surgical skills and identify surgical errors [12].

Opinion on AI

Two of the studies included surveys to evaluate the perception of AI in the medical field. Simone et al. reported that emergency surgeons expressed willingness to contribute to the development of algorithms that can be used in emergency and trauma surgery [11]. Vedula et al. surveyed surgeons and engineers in 2023 regarding the trajectory of AI in medicine over the next two, five, and 10 years. They found that within two years, AI should be able to recognize anatomy in the surgical videos and provide immediate feedback on surgical performance. Within five years, it is expected that AI will be able to feedback overlay images in real time to display surrounding anatomical structures, guide surgeons on exposure of the surgical field and guide surgeons on optimal use of instruments/devices. Within 10 years, they anticipate that AI will enable intraoperative navigability using video, kinematics, and other imaging data for multiple procedures, detect intraoperative error, provide guidance on the next best step to address an intraoperative error or complication, grade difficulty of surgical procedure [15].

Discussion

Five articles discussed intraoperative guidance, four articles discussed surgical education and training and four others were survey based. No study has gone as far as to prove that we can safely rely on AI autonomous action. The initial step in establishing autonomus action would be to establish that AI is capable of recognizing intraoperative steps in real time and identifying the next step, which has been proved in several studies on a small scale.

AI is likely to play a significant role in surgical education in several different ways. Firstly, by creating a virtual reality inspired by real images from real cases for surgical trainees to gain experience. Secondly, AI can be used to provide feedback for the trainees to improve their skills. Furthermore, this can be used as an objective skills assessment for surgical programs to assess their trainees. This not only applies to surgical trainees but also experienced surgeons where they can receive objective feedback in real time following real surgeries.

Ethical Considerations in the Development of AI

Key ethical considerations include data ownership, privacy, confidentiality and informed consent. The datasets necessary for machine learning come from real patients who have not provided consent for the data to be used in such a way. It is also reasonable to question who will have access to such data as numerous experts and third parties are required to provide annotations for machine training. Moreover, it is crucial to ensure that this data will be protected. If these obstacles are overcome, a validation process for the use of AI intraoperatively must be developed without compromising patient care; however, no such validation process currently exists.

Limitations

Several challenges were identified across multiple articles. For machine learning to occur, a large dataset is required, meaning that millions of hours of intraoperative images and videos need to be shared with the computer. This is easier in other domains of medicine such as radiology where millions of images exist to be processed by the computer [6,17,18]. This is more feasible as laparoscopic surgeries depend on visualization using the camera, however, the views are not comprehensive for the entire anatomy of the abdomen. Furthermore, each surgical procedure will need its own dataset, especially when it comes to general surgery where there is a lack of fixed, easily identifiable landmarks and it is more likely to have some variaton in anatomy [17].

The cost of such operations-from acquiring data to training the machine and validating its abilities-cannot be accurately estimated, but it likely requires substantial funding from multiple sources. Finally, should all these challenges be addressed, the question of responsibility remains: who is liable when the machine makes a mistake? [18].

## Conclusions

The current literature indicates that AI has the ability to track hand movements, identify anatomic landmarks and identify the next operative steps in real time. Furthermore, AI may play a significant role in surgical education through evaluating surgical skills and creating simulated operations for trainees to hone their skills. There are several limitations including ethical concerns, costs and feasability and finally the question or responsibility. Nonetheless, this area of AI holds great promise and warrants careful attention.
